# Medical Department of the University of Buffalo

**Published:** 1857-02

**Authors:** 


*Medical Department of the University of Buffalo.*—Before another number, the course of lectures at this institution will be concluded. The class of the present session is larger than for several years before, and the intercourse between teachers and pupils has been more than usually pleasant, although without events. The class will compare well in point of talent with any we have known. The commencement will be held on Wednesday, February 25th. The usual course in respect to examinations will be pursued, viz., an examination before the entire faculty on Tuesday; another, public, before the curators, on Wednesday, and the conferring of degrees, on Wednesday evening. Prof. Austin Flint will give the valedictory.

